# Early presymptomatic cholinergic dysfunction in a murine model of amyotrophic lateral sclerosis

**DOI:** 10.1002/brb3.104

**Published:** 2013-02-17

**Authors:** Caty Casas, Mireia Herrando-Grabulosa, Raquel Manzano, Renzo Mancuso, Rosario Osta, Xavier Navarro

**Affiliations:** 1 Group of Neuroplasticity and Regeneration Department of Cell Biology, Physiology and Immunology Institute of Neurosciences, Universitat Autònoma de Barcelona, and Centro de Investigación Biomédica en Red sobre Enfermedades Neurodegenerativas (CIBERNED)Bellaterra, Spain; 2 Laboratory of Genetic Biochemistry (LAGENBIO-I3A) Aragón Institute of Health Sciences, Universidad de ZaragozaZaragoza, Spain

**Keywords:** Amyotrophic lateral sclerosis, ChAT, MHC-I, motoneuron, presynaptic boutons, Tdp-43

## Abstract

Sporadic and familiar amyotrophic lateral sclerosis (ALS) cases presented lower cholinergic activity than in healthy individuals in their still preserved spinal motoneurons (MNs) suggesting that cholinergic reduction might occur before MN death. To unravel how and when cholinergic function is compromised, we have analyzed the spatiotemporal expression of choline acetyltransferase (ChAT) from early presymptomatic stages of the SOD1^G93A^ ALS mouse model by confocal immunohistochemistry. The analysis showed an early reduction in ChAT content in soma and presynaptic boutons apposed onto MNs (to 76%) as well as in cholinergic interneurons in the lumbar spinal cord of the 30-day-old SOD1^G93A^ mice. Cholinergic synaptic stripping occurred simultaneously to the presence of abundant surrounding major histocompatibility complex II (MHC-II)-positive microglia and the accumulation of nuclear Tdp-43 and the appearance of mild oxidative stress within MNs. Besides, there was a loss of neuronal MHC-I expression, which is necessary for balanced synaptic stripping after axotomy. These events occurred before the selective raise of markers of denervation such as ATF3. By the same time, alterations in postsynaptic cholinergic-related structures were also revealed with a loss of the presence of sigma-1 receptor, a Ca2+ buffering chaperone in the postsynaptic cisternae. By 2 months of age, ChAT seemed to accumulate in the soma of MNs, and thus efferences toward Renshaw interneurons were drastically diminished. In conclusion, cholinergic dysfunction in the local circuitry of the spinal cord may be one of the earliest events in ALS etiopathogenesis.

## Introduction

Dysfunction of the cholinergic system is a common feature in Alzheimer's disease, Huntington's disease, and amyotrophic lateral sclerosis (ALS). In the formers, little is known surprisingly about the implication of cholinergic dysfunction with disease etiopathogenesis. In ALS, cholinergic diminution has been presumed to be associated in late stages, with motoneuron (MN) loss. Choline acetyltransferase (ChAT, acetyl CoA: choline O-acetyltransferase, EC 2.3.1.6), the enzyme responsible for the biosynthesis of acetylcholine, is the most specific indicator for monitoring the functional state of cholinergic neurons in the central and peripheral nervous systems. ChAT mediates the reaction involving the transfer of an acetyl group from acetyl coenzyme A to choline to form acetylcholine (ACh) at the synaptic endings of cholinergic neurons. ChAT is synthesized in the perikaryon of cholinergic neurons, and a minor proportion is transported by fast axonal transport, mainly mediated by kinesins (Ray et al. 1999). At the synaptic terminals, ACh is synthesized in the cytoplasm and stored into synaptic vesicles by the vesicular acetylcholine transporter (VAChT).

ALS selectively affects MNs in the brain and spinal cord, resulting in progressive weakness and wasting of muscles. Histopathologically, there is loss of upper MNs in the cerebral motor cortex, and prominent loss of lower cholinergic MNs in the motor nuclei of the brainstem and the anterior horn of the spinal cord (Cleveland and Rothstein 2001). Both sporadic and familiar cases of ALS present a marked reduction in ChAT activity in the anterior horn of the spinal cord (Wang et al. 1997). Far from being only a reflection of neuronal loss, microassay analysis of ChAT activity of single neurons has demonstrated that large, preserved neurons at an early stage of the disease show lower ChAT activity than control neurons (Kato 1989; Oda et al. 1995). Morphologic studies have also demonstrated a marked loss of ChAT mRNA in spinal cord of ALS patients by in situ hybridization (Virgo et al. 1992). These findings suggested that low expression of ChAT in the spinal cord may represent an early abnormality in the pathogenesis of ALS (Oda 1999), starting long before MN death. However, the causes of sporadic ALS remain obscure. Since the discovery of the genetic linkage of mutations in superoxide dismutase 1 (SOD1) gene with familial ALS patients, one of the highlighted putative mechanisms is that degeneration of MNs is closely linked to involvement of SOD1 in both sporadic and familial cases (Bosco et al. 2010). It is proposed that the occurrence of misfolded SOD1 triggers a cascade of neurodegeneration by “gains-of-function” through activation of glutamate-mediated excitotoxicity, which induces an uncontrolled increase of intracellular calcium concentration (de Carvalho and Swash 2011). Data regarding cholinergic activity in animal models carrying SOD1 mutations are mainly reported linked to MN loss in the symptomatic phase (Crochemore et al. 2005; Alves et al. 2011).

Nevertheless, a question that remains to be solved is how and when cholinergic function is compromised along the neurodegenerative process. In order to answer these questions, we have analyzed the spatiotemporal expression of ChAT, considering local cholinergic circuitry, efferences, and afferences, within the spinal cord from early presymptomatic until symptomatic stages of an ALS mouse model. The results obtained highly the importance of the performance of longitudinal studies to unravel the etiopathogenesis of ALS.

## Material and Methods

### Animals

Experiments were performed in transgenic mice carrying the mutation G93A in SOD1 gene and in nontransgenic wild-type (WT) littermates considered controls. SOD1^G93A^ high copy mice (Tg[SOD1-G93A]1Gur) were obtained from the Jackson Laboratory (Bar Harbor, ME), with B16xSJL background. These mice were bred and maintained as hemizygotes by mating transgenic males with F1 hybrid (B6SJLF1/J) females obtained from Charles River Laboratories (Belgium). Animals were bread at the Animal Supply Services, Unidad Mixta de Investigación, University of Zaragoza, and were cared for and handled in accordance with the guidelines of the European Union Council (86/609/UE) and Spanish regulations (BOE 67/8509-12; BOE 1201/2005) on the use of laboratory animals. Experimental procedures were approved by the local Ethics Committee of the Universitat Autònoma de Barcelona. Transgenic mice were identified by polymerase chain reaction amplification of DNA extracted from the tail. Studies were performed in groups of 1-, 2-, and 3-month-old female mice (*n* = 8 each). One- and 2-month-old SOD1^G93A^ mice are considered to be in early and adult presymptomatic stages of disease, respectively, whereas 3-month-old mice had an early symptomatic phenotype by behavioral (Chiu et al. 1995) and electrophysiological testing (Mancuso et al. 2011).

### Immunohistochemistry

Animals were anesthetized with sodium pentobarbital (50 mg/kg i.p.), and perfused transcardially with phosphate buffered saline (PBS), followed by 4% paraformaldehyde in 0.1 mol/L PB, pH 7.4 at 4°C. The spinal cord was removed, divided into lumbar, thoracic and cervical segments, postfixed for 24 h and cryopreserved in 30% sucrose in PBS at 4°C. Transverse sections (40 μm thick) were obtained with a cryostat (Leica, Heidelberg, Germany) individually placed on 96-well plates in Olmos solution and stored at −20°C. The sections were distributed in 50 series of five sections each, and each series was prepared for immunohistochemical analysis by blocking with 10% bovine serum, 0.3% Triton X-100 in tris-buffered saline for 1 h at room temperature, followed by incubation with different combinations of up to three primary antibodies against synaptotagmin (clone Mab48, Developmental Studies Hybridoma Bank, IA), nitrotyrosine (Millipore, Bedford, MA), human HCA-ABC antigen (MHC-I, DAKO, Glostrup, Denmark), MHC-II-APC (eBiosciences, SanDiego, CA), Iba1 (Wako, Tokyo, Osaka, Japan), sigma 1 Receptor (Sig1-R, Santa Cruz Biotechnologies, Santa Cruz, CA) and ChAT (Millipore) overnight at 4°C. After washes, sections were incubated for 1 day at 4°C with biotinylated secondary antibodies (Vector, Burlingame, CA, 1: 200) with Cy-2, Cy-3, or Cy-5 conjugated donkey anti-rabbit, anti-mouse, or anti-goat IgGs antibodies (Jackson Immunoresearch, West Grove, PA, 1:200). Slides were counterstained with DAPI (4′,6-diamidino-2-phenylindole) (Sigma, St Louis, MO, 1: 1000) and mounted with Fluoromount (SouthernBiotech, Birmingham, AL). Omission of the primary antibodies resulted in no detectable staining. At lumbar levels, the analysis was focused in MNs from L4–L5 segments that provide innervation to hindlimb muscles. Sections from different time points of transgenic and control animals were processed in parallel for immunohistochemistry and data represent an accumulation of different day performances. Confocal microscope examinations were performed with a Leica TCS SP2 AOBS laser scanning confocal system (Leica). All MNs were analyzed in a *z*-plane containing the nucleus and captured using the FV10-ASW 1.7 Viewer software. Confocal images were obtained using two separate photomultiplier channels, either concurrently or in separate runs, and were separately projected and merged using a pseudocolor display showing green for Cy2, red for Cy3, magenta for Cy5, and blue for DAPI. When densitometric analysis was performed, images of the ventral area of the spinal cord were taken under the same exposure time, sensibility, and resolution for each marker analyzed, with the aid of a digital camera (Olympus DP50) attached to the microscope (Olympus BX51). The microphotographs were transformed to a gray scale and analyzed using ImageJ software. Immunoreactivity was assessed by calculating the integrated density, after defining a threshold for background correction. The integrated density of a region of interest (ROI), defined as the area above the threshold for the mean density minus the background, was measured. The ROIs were selected on the gray matter of the ventral horn and had an area of 4900 μm^2^ for ChAT-stained ventral horn MNs and 1300 μm^2^ for cholinergic ChAT-stained interneurons. The average cell size was similar between the genotypes and stable over the observation period.

### Quantification of ChAT-positive synaptic boutons

The quantitative evaluation of synaptic boutons was carried out by confocal analysis. Projection of section images (0.68 μm) obtained from a *Z*-plane screening of each sample was maximized to obtain single image of uniform thickness (10 μm) that contained the whole motoneuronal soma. We counted both the number of large ChAT-positive varicosities and the synaptotagmin-positive large terminals apposing the soma of the MNs per perimeter using the Metamorph 2.0 software. The evaluation included 24–36 MNs per spinal cord at L4 level (in 3–4 animals per phenotype).

### Molecular analysis

Half lumbar spinal cord from WT and transgenic animals at different ages were obtained and processed for either RNA or protein analysis. Protein was obtained by collecting the tissue in lysis buffer (20 mmol/L HEPES [4-(2-hydroxyethyl)-1-piperazineethanesulfonic acid buffer], 250 mmol/L sucrose, 1 mmol/L EGTA [ethylene glycol tetraacetic acid], 1 mmol/L EDTA [ethylenediaminetetraacetic acid], pH 7.4). Lysates were homogenated with Pellet pestle (Sigma, St Louis, MO) and spin at 800*g*. An equal amount of protein (20 μg/lane) was resolved in 10% SDS-PAGE (sodium dodecyl sulfate polyacrylamide gel) and electrotransferred to PVDF (polyvinylidene difluoride) membranes (Millipore). Membranes were blocked with 6% nonfat dry milk in PBS (140 mmol/L NaCl, 2.7 mmol/L KCl, 4.3 mmol/L Na_2_HPO_4_·H_2_O, and 1.5 mmol/L KH_2_PO_4_) for 1 h at room temperature and incubated overnight with the corresponding primary antibody, ChAT (1:1000, Chemicon), or actin (1:10,000, Sigma). After several washing, membranes were incubated for 1 h with an appropriate secondary antibody conjugated with horseradish peroxidase (1:3000, anti-mouse-HRP; Dako, Denmark) and anti-rabbit-HRP (Invitrogen, Carlsbad, CA). Blots were developed using a chemiluminiscent mix 1:1 (0.5 mol/L luminol, 79.2 mmol/L *p*-coumaric acid, 1 mol/L Tris-HCl pH 8.5 and 8.8 mol/L hydrogen peroxide, 1 mol/L Tris-HCl pH 8.5) and exposed to enzymatic chemiluminiscence (ECL) films (Amersham Pharmacia Biotech, Buckinghamshire, UK). The apparent molecular weight of proteins was determined by calibrating the blots with prestained molecular weight marker (All Blue, Pierce). Densitometry was carried out using ImageJ program.

The other half of the tissue was obtained in RNAlater (Qiagen, Valencia, CA) and processed with Quiagen easy kit following manufacturer instructions. One microgram of RNA was reverse transcribed using 10 mmol/L DTT, 200 U Superscript II RNase H reverse transcriptase (Invitrogen, Foster City, CA), 10 U RNase Out Ribonuclease Inhibitor (Invitrogen) and 1 mol/L oligo(dT), 1 mol/L of random hexamers (BioLabs, Beverly, MA). The reverse transcription cycle conditions were 25°C for 10 min, 42°C for 1 h, and 72°C for 10 min. The primers used for RT- and real-time polymerase chain reaction (PCR) were *ChAT* (F, 5′-TGGATGGTCCAGGCACTGGAGACC-3′; R, 5′-GTCA TACCAACGATTCGCTCCATTCA-3′) and glyceraldehyde-3-phosphate dehydrogenase (*Gapdh*) (F, 5′-ACCACCATGGAGAAGGCCGG-3′; R, 5′-CTCAGTGTAGCCCAAGATGC-3′). PCR products yielded fragments smaller than 150-bp length. Real-time PCR (ABI prism 7700 detection system, PE Applied Biosystems, Foster City, CA) was performed using Brilliant III Ultra-Fast SYBR® Green qPCR master mix (Agilent Technologies, Santa Clara, CA, USA). We previously fixed the optimal concentration of the cDNA to be used as template for each gene analysis to obtain reliable C_T_ (threshold cycle) values for quantification. Four samples were used per condition and each sample was run in triplicate. The thermal cycling conditions were 50°C for 2 min, 95°C for 10 min, and 40 cycles of 95°C for 15 sec, 60°C for 1 min. C_T_ values were obtained and analyzed with the ABI prism 7700 SDS Software. Fold change in gene expression was estimated using the C_T_ comparative method (2^−ΔΔCT^) normalizing to *Gapdh* C_T_ values and relative to the average of control samples. Melting curves confirmed amplification of solely one PCR product for all qPCRs.

### Statistical analysis

Data are expressed as the mean ± SEM. Comparisons between groups of mice of different ages were made by one-way analysis of variance (ANOVA) with post hoc Dunnett's multiple comparison test for IHC analysis using GraphPad Prism 5.01 software. For qPCR analysis, it was used a nonparametric Mann–Whitney test. Statistical significance was set at *P* < 0.05. The number of analyzed MNs and number of animals are indicated in the results section, as well in the figure legends.

## Results

### ChAT immunoreactivity

In the WT mice at all ages analyzed, normal ChAT expression was located in the perikaryon, nucleus and processes as well as in presynaptic terminals apposed onto MNs at the ventral horn of the spinal cord. We also observed ChAT within cholinergic interneurons placed around the central canal (lamina X) and extended to the lateral edge in the gray matter.

**Figure 1:**
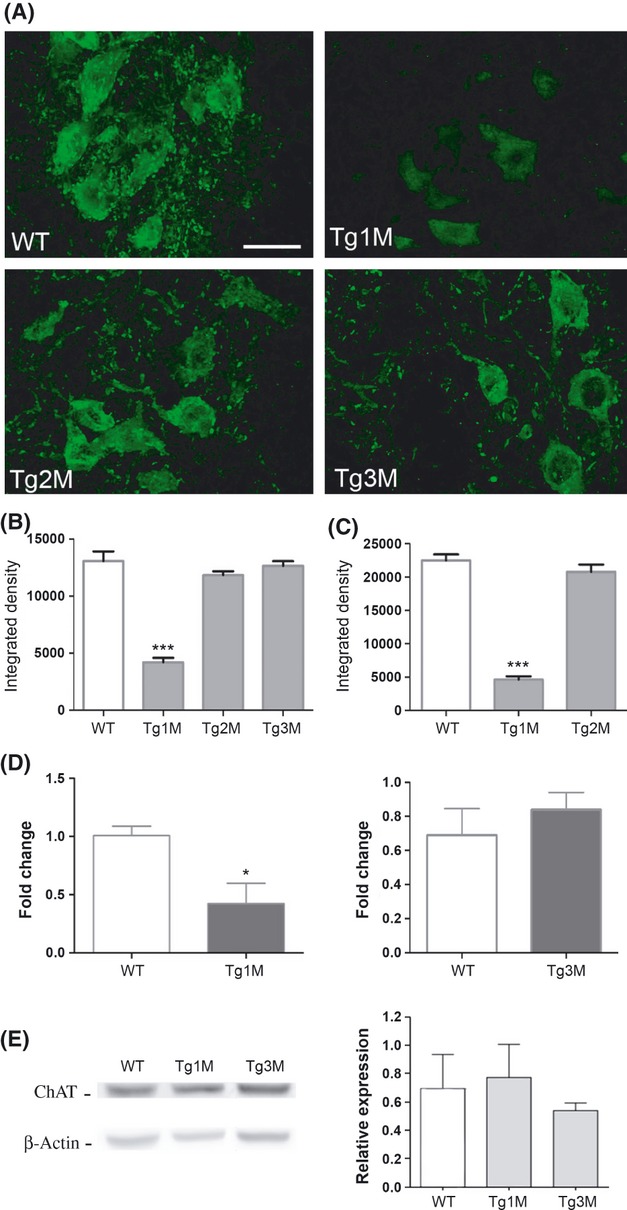
Early transient ChAT reduction in spinal MNs of transgenic SOD1 mice. (A) Immunofluorescent microphotographs showing ChAT content in MNs at the L4–L5 spinal cord ventral horn of nontransgenic wild-type (WT) littermates or transgenic SOD1 mice (Tg) at 1, 2, and 3 months of age. Scale bar, 50 μm. (B, C) Bar graphs representing average ± SEM of the integrated density of ChAT content within a ROI of 4900 μm^2^ in single MNs at lumbar (B) and thoracic levels (C) of the spinal cord of WT and Tg SOD1 mice at different ages (*n* = 4 animals/group, *n* = 13–65 MNs per animal). A transient reduction was observed in Tg mice by 1 month of age. ***P < 0.0001. (D) *ChAT* mRNA transcript analysis by quantitative PCR of lumbar spinal cord at 1 and 3 months of age. Bars represent the average fold change respect to their controls and *gapdh* mRNA expression, *n* = 4, *P < 0.05. (E) Western blot results of murine ChAT protein content in the same samples and bar graph representing the content average respect to the β-actin.

When analyzing its temporal expression, we observed a transient reduction in CHAT immunoreactivity within the soma of MNs in transgenic mice carrying the mutation G93A in SOD1 gene (SOD1^G93A^) compared with the WT littermates (Fig. 1). We analyzed separately lumbar and thoracic segments as this mouse model is known to present a progressive caudal to rostral degeneration of the MNs (Gurney et al. 1994). We found that ChAT immunoreactivity was significantly decreased in ventral MNs at 1 month of age but close to normal at 2 and 3 months (Fig. 1A–C). This reduction was observed in practically all MNs located either lateral or medially in the ventral horn at different thoracic (decrease of 80 ± 2% at 1 month, *n* = 13–31) and lumbar (69 ± 3%, *n* = 13–64) levels. We also observed that the ChAT content was normally localized within the whole MN soma, including the nucleus, in the 1-month-age SOD1^G93A^ mice, but it seemed to be mainly located in the cytoplasm in the 3-month-age mice observed by confocal analyses. Besides, CHAT-labeled processes were sharper and shorter from 2 months of age (data not shown). We performed Western blot analysis and quantitative PCR to analyze the expression level of ChAT protein and transcript, respectively, in lumbar sections from 1 and 3 month of age animals. We observed no significant differences at protein level at any time point between transgenic and control littermate animals; however, it was a marked reduction in the transcript of ChAT in the 1-month-age SOD1^G93A^ mice (Fig. 1D and E).

**Figure 2:**
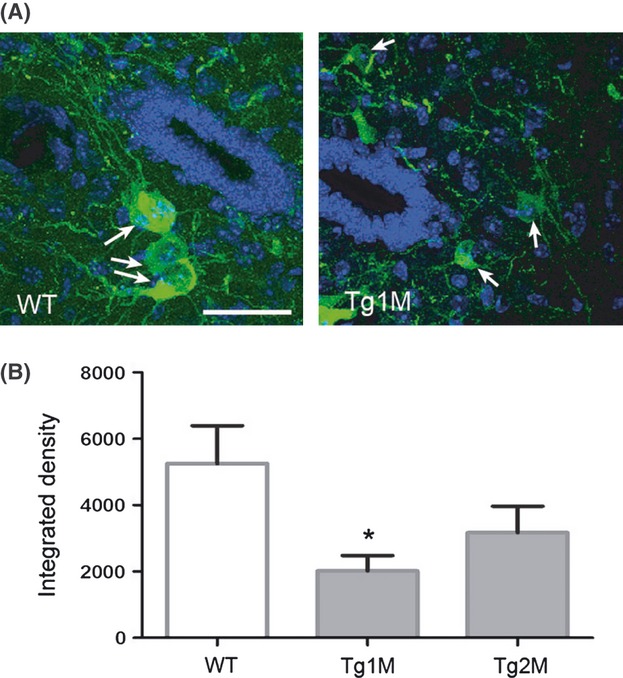
Cholinergic interneurons have early decrease of ChAT in the ALS mouse model. (A) Representative immunofluorescent microphotographs showing ChAT labeling in cholinergic interneurons (green) contrasted with cellular nuclear staining with DAPI (blue), near the central canal at the lumbar spinal cord of wild-type (WT) and Tg SOD1 mice. Arrows point some cholinergic interneurons. Scale bar, 50 μm. (B) Quantification of ChAT integrated density of staining (ROI of 1300 μm^2^, around interneuronal soma) in these interneurons are represented by mean ± SEM in a bar graph showing reduction most marked in Tg animals of 1 month of age (*n* = 4 animals/genotype, *n* = 13 neurons per animal). *P < 0.05.

In order to investigate whether it was a general effect affecting the production of ChAT independently of the type of neuron or it was specific for MNs, we analyzed also the cholinergic interneurons present in lamina X that innervate MNs at the lumbar level. We observed that cholinergic interneurons presented also a reduction in ChAT content within their soma (61 ± 8%, *n* = 13) at 1 month of age, which increased at 2 months but it was still significantly lower than in WT mice (Fig. 2).

These results indicated that there is a generalized, early, and transient reduction in ChAT content in the soma and processes of cholinergic neurons, both MNs and interneurons of the spinal cord, in SOD1^G93A^ mice at 30 days of age. This decline persists in the processes but not in the soma of MNs in older transgenic mice.

### Quantitation of ChAT-positive boutons

**Figure 3:**
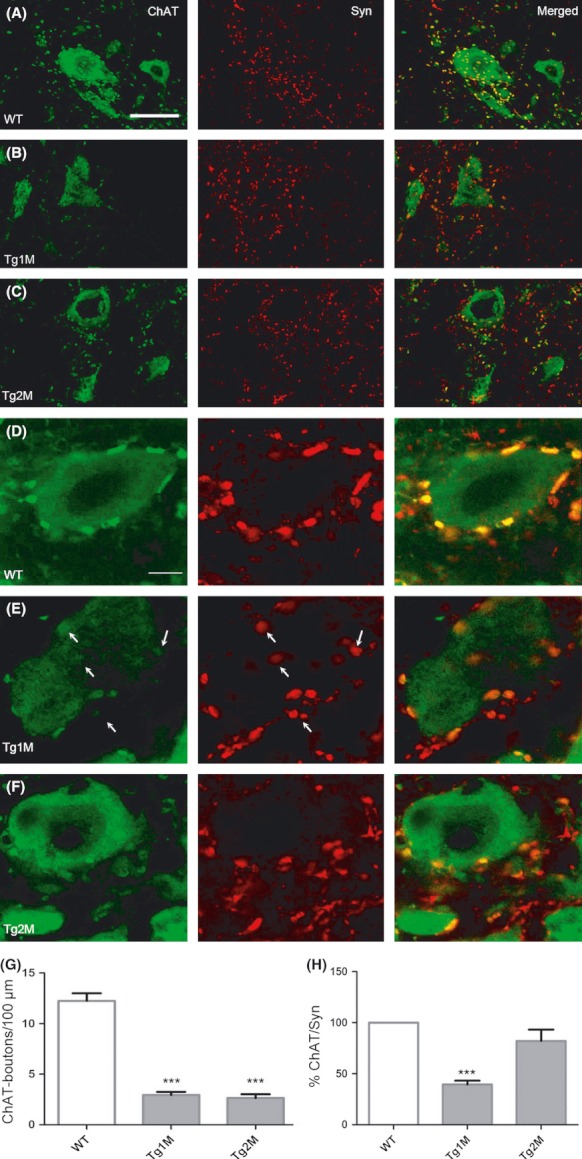
Early reduction of ChAT content precedes loss of large synaptic boutons. (A–C) Representative imunofluorescent microphotographs showing ChAT (green), synaptotagmin (Syn, red), and merged images (yellow–orange staining in colocalization) around MNs at the L4–L5 ventral horn of WT and transgenic mice of 1 and 2 months of age. Note an overall reduction in ChAT-positive dots around MNs in (B), and a change in the quality of Syn dots with decreased density of large ones apposing MNs in (C). (D–F) High magnification microphotographs of MNs from WT and transgenic mice of different ages showing synaptic boutons apposing to MNs. Note in (D), that all ChAT-positive synaptic boutons matched with synaptotagmin labeling while in (E), large Syn-positive synaptic boutons have absent or reduced ChAT content (arrows pointing some of them). Note that some ChAT-positive terminals seem to be larger than in the WT mice. In (F), Syn-positive dots were reduced in density and most of the largest ones were ChAT positive. Scale bar, A–C, 50 μm; D–F, 10 μm. (G) Quantification of the number of ChAT-positive boutons apposing MN somata per 100 μm of membrane perimeter (mean ± SEM). (H) Percentage of large Syn-boutons that are also ChAT positive (mean ± SEM). ***P < 0.0001 two-way analysis of variance, post hoc Dunnet's test.

As mentioned, ChAT was also observed in cholinergic terminals that contact onto spinal MNs, which belong to either recurrent axonal collaterals of interconnections between MNs (Cullheim et al. 1977) (Lagerback et al. 1981) or innervation by cholinergic interneurons. These inputs influence the MN excitatory and inhibitory balance, which is altered in ALS. Those terminals apposing MN somata are named C-boutons and represent one of the largest terminals around their perimeter (3–7 μm in cat) (Arvidsson et al. 1997). In order to analyze the ChAT content in these terminals, we counted ChAT-labeled boutons apposed to MNs at L4–L5 in WT and SOD1^G93A^ mice at 1 and 2 months of age. We found a marked decrease (76%) in SOD1^G93A^ mice already from 1 month of age (Fig. 3). No statistical differences were found between nontransgenic animals of 1 or 2 months of age. In order to correlate the presence of ChAT content with the structural existence of the synaptic terminal, we counted also the large synaptotagmin-positive boutons apposed to MNs somata (putatively C-boutons). We found that their density was normal at 1 month of age (10.2 ± 2 boutons/100 μm) although only 39% of these boutons were ChAT positive (Fig. 3). In contrast, the number of large synaptotagmin-positive boutons decreased (5 ± 0.6 boutons/100 μm) by 2 months of age and most of them were ChAT-positive boutons (82%). These results indicated that the content of ChAT within large boutons progressively diminished from 1 month of age and the frequency of these cholinergic terminals tended to be reduced in the 2-month SOD1^G93A^ mice.

**Figure 4:**
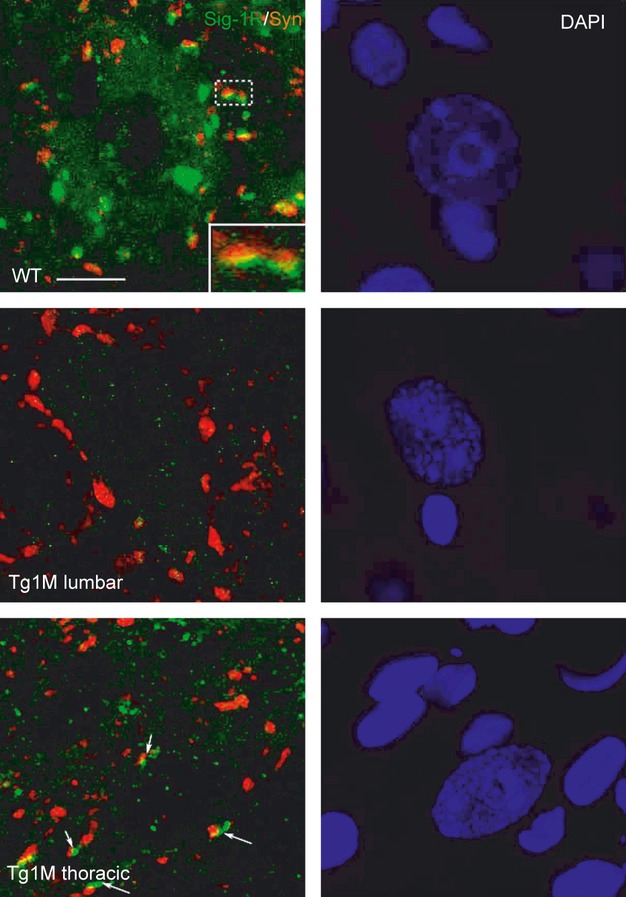
Early loss of Sigma 1 receptor expression in lumbar MNs from transgenic SOD1^G93A^ mice at 1 month of age. Representative confocal overlayed microphotographs showing Sig1-R (green) localized in the postsynaptic sites within the MNs, and synaptotagmin staining of presynaptic terminals (red) (left panels). The inset of top-left panel shows a detail of close localization of both markers at higher magnification. In the right panels, microphotographs show DAPI staining to reveal the presence of the MN nucleus. WT panel show a representative MN image from lumbar site although thoracic site presented the same pattern (not shown). Note the absence of Sig1-R immunoreactivity in lumbar MNs but still presence in thoracic MNs (arrows) of the spinal cord from mutant transgenic mice of 1 month of age. Scale bar, 50 μm.

In the postsynaptic membrane of the MN, beneath some cholinergic presynaptic boutons, there is a subsynaptic cistern. The cistern is thought to be continuous with the rough endoplasmic reticulum (ER) and directly associated with the function of the synapse (Nagy et al. 1993). In these cisterns, the sigma 1 receptor (Sig1-R) is present to buffer Ca^2+^ entry overload (Mavlyutov et al. 2010). We found Sig-1R immunoreactivity at close proximity of the synaptic clefts in a spotty appearance in MNS of WT mice, but it was absent in lumbar MNs from SOD1^G93A^ mice of 1 month of age (Fig. 4). Curiously, it was still present in thoracic MNs of the same animals although in smaller spots than in the WT (Fig. 4).

**Figure 5:**
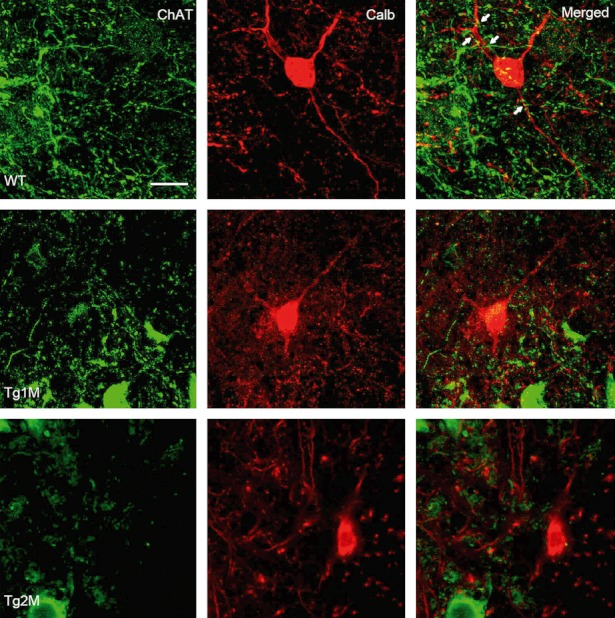
Cholinergic inputs on Renshaw neurons are reduced in transgenic SOD1^G93A^ mice. Representative confocal overlayed microphotographs showing ChAT immunolabeling (green, left panels), calbindin labeling (red, middle panels) to reveal Renshaw interneurons and merged images (left panels) from the ventral horn of lumbar spinal cord from wild-type and transgenic SOD1 mice. Arrows point some cholinergic terminals on dendrites of Renshaw neurons that are hardly detected in transgenic mice. Scale bar, 50 μm.

Further evidences of cholinergic alterations were observed in the local circuitry established between MNs and Renshaw interneurons in the ventral horn. We labeled Renshaw cells with anticalbindin and observed the cholinergic boutons onto their surface. The presence of cholinergic terminals along their processes was diminished form the 1-month-old SOD1^G93A^ mice (Fig. 5). Also note the lack of ChAT staining in the processes efferent from MNs.

In conclusion, these data indicate that ChAT activity may be reduced in the synaptic terminals from very early in the presymptomatic stage of the SOD1^G93A^ mice. This abnormality affects both afferences and efferences onto and from MNs, respectively, that participate in the local spinal motor circuitry.

### Synaptic stripping

**Figure 6:**
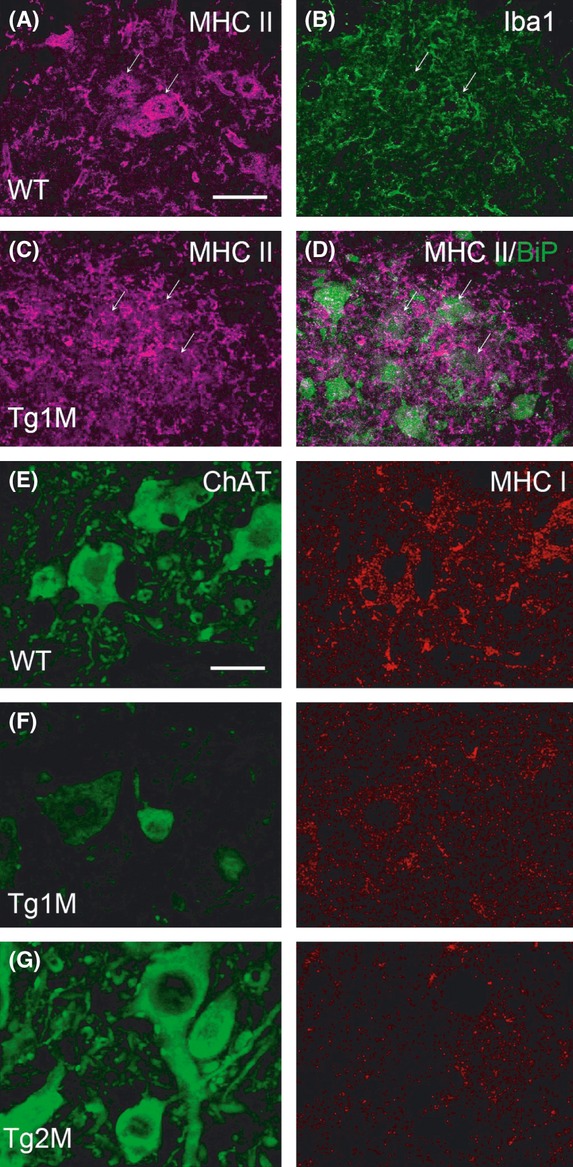
Early reduction of MHC-II and MHC-I expression within MNs versus increment of MHC-II-positive surrounding microglia in lumbar ventral horns of transgenic SOD1^G93A^ mice from 1 month of age. (A, B) Representative confocal projection of microphotographs showing MHC-II expression (magenta, A) in both MNs and microglial cells. MNs are recognized by its form and size (arrows) and microglia is recognize by colabeling with IbaI showed in the adjacent panel (green, B) at the lumbar ventral horn of WT mice of 1 month of age. (C, D) In the transgenic SOD1^G93A^ mice of the same age (tg1M), abundant microglia expressing MHC-II is seen around MNs (arrows) in (C). In (D), a merged image from (C) is shown to reveal the presence of MNs by its expression of a constitutive and ubiquitous chaperone (BiP, green in D). (E, F) Representative confocal overlayed microphotographs showing ChAT immunolabeling (green, left panels) and the progression of MHC-I labeling (red, right panels) in MNs from wild-type and transgenic SOD1^G93A^ mice of 1 and 2 months. WT animals from 1 and 2 months of age present the same pattern as in (E). Note progressive reduction of MHC-I within the MNs of transgenic mice. Scale bar, A–D, 200 μm; E–G, 50 μm.

To shed light about the reduction of large synaptotagmin-positive boutons apposed to the MN soma, we examined the presence of perineuronal microglia. It is known that microglia is actively involved in the processes of synaptic stripping that normally occur in response to peripheral axotomy (Blinzinger and Kreutzberg 1968). We used Iba1 as a marker for microglia and observed the expression of a major histocompatibility complex II (MHC-II) protein that is overexpressed in phagocytic microglia (Bo et al. 1994). In the WT mice, independently of the age, MHC-II was observed in both sparse microglia and within the MN soma (Fig. 6A and B). In the SOD1^G93A^ mice, already from 1 month of age, there was a loss of MHC-II neuronal expression concurrent with abundant MHC-II-positive microglia surrounding MNs (Fig. 6C and D).

Another member of the major histocompatibility complex, MHC-I, is implicated in the cross-talk between microglia and MNs for selective synaptic stripping during development, plasticity, and regeneration (Huh et al. 2000). We observed that MHC-I was expressed within MN soma and processes with a dotted pattern in WT mice. In the SOD1^G93A^ mice, MHC-I expression seemed to be weaker than in WT, and it was practically absent at 2 months of age (Fig. 6E–G).

The presence of aggregated MHC-II-positive phagocytic microglia surrounding MNs that present themselves a downregulation of both MHC-I and MHC-II suggested that altered synaptic stripping may be an early event in the pathogenesis of MN degeneration.

### Relation with oxidative and ER stress

In order to link the presence of two of the main processes involved in ALS-associated neurodegeneration, oxidative, and ER stress, with our observations regarding ChAT altered expression, we analyzed the concurrence of these events.

**Figure 7:**
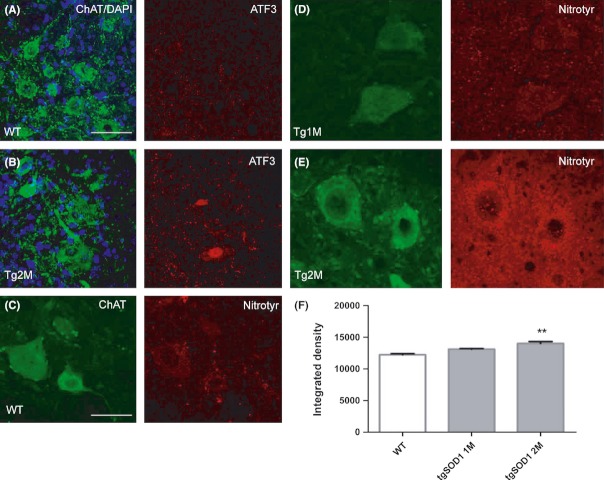
Nitro-oxidation and endoplasmic reticulum stress appear later than cholinergic alterations. (A, B) Representative confocal microphotographs showing ChAT labeling (green) contrasted with nuclear staining with DAPI (blue) in left panels and ATF3 labeling (red) of the same cells in right panels. Note that there is no immunoreactivity for ATF3 in WT mice but strong nuclear presence in some MNs of the ventral horn of transgenic mice by 2 months of age. (C–E) Nitrotyrosine labeling (red) of the ventral horn spinal cord from WT and transgenic mice of 1 and 2 months of age (right panels). Note the progressive accumulation of nitrotyrosine in the parenchyma and within the MNs. Scale bar, A–B, 100 μm; C–E, 50 μm. (F) Bar graph representing mean ± SEM of integrated density of nitrotyrosine labeling in the ventral horn of the spinal cord from WT and transgenic SOD1^G93A^ mice.**P < 0.01 two-way analysis of variance, post hoc Dunnet's test.

It has been observed that SOD1^G93A^ mice start to show detachment of neuromuscular junctions as early as 47 days of age (Pun et al. 2006). Besides, this denervation triggers the overexpression of ER stress markers in the MNs (Saxena et al. 2009), being ATF3 the one appearing first in the SOD1^G93A^ mice. ATF3 is downstream ATF4 that forms part of one the main branches of the response triggered by ER stress. On the other hand, denervation and axotomy often shut ChAT expression off in the MNs (Penas et al. 2009). Thus, we were interested in linking these early events to sequence its order of appearance and establish a putative causal link. When analyzing ATF3 expression by immunohistochemistry, we observed that it was notably increased within the nucleus of only selective spinal MNs from 2-month-old SOD1^G93A^ mice, but it was completely absent in animals at 1 month of age (Fig. 7). Therefore, cholinergic dysfunction appears to precede ER stress in the MNs.

Considering that SOD1 manages the accumulation of reactive species, and oxidative stress has been related to the neurodegenerative process in ALS (Basso et al. 2009), we analyzed the time course of accumulation of nitrosative reactive species. We analyzed nitrotyrosine levels in gray and white matter of the spinal cord, as nitrosative reactive species have been early detected in the SOD1^G93A^ mice. Besides, high levels of nitrotyrosine activate microglia to initiate synaptic stripping (Moreno-Lopez et al. 2011). The staining with antinitrotyrosine was practically absent in WT spinal cord; however, we observed that nitrotyrosine levels progressively increase in the gray matter and within the MN soma in SOD1^G93A^ mice, reaching significantly higher values at 2 months of age (Fig. 7).

These data suggested that cholinergic alterations may occur earlier than peripheral neuromuscular detachment and consequently induced ER stress, but in parallel to the initial accumulation of oxidative reactive species.

### Tdp-43

Finally, considering that Tdp-43, also linked to ALS etiopathogenesis, is involved in multiple steps of RNA metabolism, including transcription, splicing, or transport of mRNA (Lagier-Tourenne and Cleveland 2010), as well as microRNA metabolism, and it has been recently shown to target ChAT mRNA as well, (Buratti et al. 2010) we wanted to analyze its expression at early presymptomatic stages in this mouse model.

**Figure 8:**
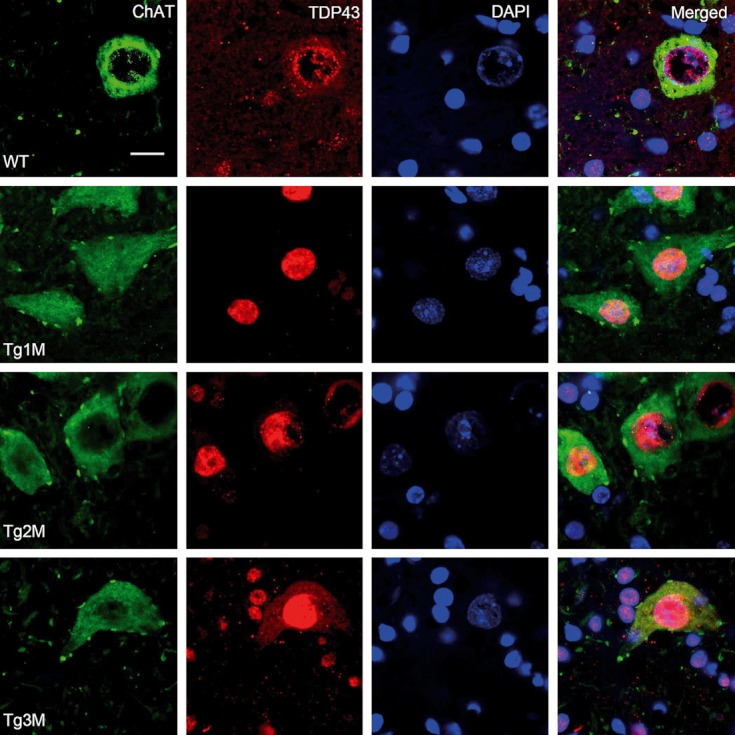
Tdp-43 is markedly accumulated in the MN nucleus of the transgenic SOD1^G93A^ mice. Representative confocal microphotographs of single sections where a MN nucleus is present (DAPI staining in blue) showing ChAT (green) and Tdp-43 (red) immunolabeling, and merged images of MNs at the ventral horn of the lumbar spinal cord from wild-type and transgenic SOD1^G93A^ mice of 1, 2, and 3 months. Note that all pictures were performed at the same conditions of exposition. Scale bar, 50 μm.

Tdp-43 was found normally present in both nucleus and cytoplasm of the MNs in WT mice. In contrast, Tdp-43 was markedly overexpressed and accumulated in the nucleus but barely detected in the cytoplasm of spinal MNs in the SOD1^G93A^ mice already at 1 month of age (Fig. 8). The same pattern was observed at 2 months. In contrast, from the symptomatic stage, by 3 months of age, Tdp-43 levels increased also in the cytoplasm of MNs and in the nucleus of surrounding glial cells within the spinal cord parenchyma.

In conclusion, both the levels and localization of Tdp-43 in all the spinal MNs are severely affected early in the presymptomatic stage in SOD1^G93A^ mice, and parallels the development of cholinergic dysfunctions.

## Discussion

Synaptic cholinergic dysfunction is a common feature of different neurodegenerative diseases, including ALS, but little is known regarding the possible relationship between ChAT abnormalities and the pathogenesis of MN degeneration. A detailed longitudinal study performed in the present work shows that there is a marked reduction of neuronal ChAT content affecting synaptic function in the local spinal cord circuitry of the transgenic SOD1^G93A^ mouse model of ALS as early as 30 days of age, an early presymptomatic stage. Timely highlighted concurrent events are Tdp-43 overexpression in the nucleus of MNs and the presence of mild oxidative stress.

Loss of cholinergic synapses was reported in ALS patients (Nagao et al. 1998) and subsequent studies of central synaptic connections of lumbar spinal MNs suggested that synaptic dysfunction precedes synaptic loss (Matsumoto et al. 1994; Sasaki and Maruyama 1994; Ikemoto et al. 2002). However, limited investigation targeting ChAT directly has been carried out on ALS animal models. In the SOD1^G93A^ model, the most studied one, ChAT activity has been analyzed either by enzyme activity determination (Crochemore et al. 2005) or by Western blot of whole spinal cord extracts (Alves et al. 2011). These studies did not reveal any abnormalities before the symptomatic phase and thus later cholinergic dysfunction was attributed to MN loss. We confirm these observations when analyzed the protein levels by Western blot of the whole lumbar spinal extract. However, by using immunohistochemical analysis of ChAT expression, which is more sensitive to demonstrate specific changes in levels and in particular cells, we show for the first time that ChAT content is clearly reduced in soma of MNs and cholinergic synaptic terminals very early, by 1 month of age, before any loss of MNs occurs. We also observed this reduction in cholinergic interneurons. These interneurons normally make synapses onto MNs (putatively cholinergic C-boutons) (Barber et al. 1984) to ensure that sufficient output is generated by MNs to drive motor behavior (Miles et al. 2007; Zagoraiou et al. 2009). Thus, reduction of ChAT levels in cholinergic interneurons and MN somata themselves contributes to the observed reduction in ChAT content in the synaptic boutons apposed to spinal MNs. Consistently, an early reduction in *ChAT* transcript content was also observed by that time suggesting that signaling changes in neuronal metabolism are implicated. Considering several early pathological events described in SOD1^G93A^ mice, we were trying to figure out the possible cause linked to this early ChAT reduction. On one hand, ALS has been proposed to be a distal axotomy type of neurodegenerative disease (Dadon-Nachum et al. 2011). In agreement with that, SOD1^G93A^ mice show detachment of neuromuscular junctions as early as 47 days of age, followed by a severe loss of motor axons in the ventral root between days 47 and 80, and electrophysiological studies revealed reduced neuromuscular responses by 2 months of age (Mancuso et al. 2011), well before α-MN cell bodies die around day 110 (Fischer et al. 2004; Dadon-Nachum et al. 2011). Furthermore, disconnected vulnerable MNs selectively overexpress markers of ER stress like ATF3 by the same time (Saxena et al. 2009). As early reduction of ChAT is a common event in damaged MNs after peripheral disconnection (Wang et al. 1997; Kitzman 2006; Penas et al. 2009), a possibility was that the reduction observed in the SOD1^G93A^ mouse model was merely due to the initial distal detachment. However, we observed that ChAT reduction occurred earlier, by 30 days of age, and before ATF3 overexpression. Besides, ChAT reduction was observed in all the MNs, and not only in the most vulnerable ones that selectively presented ATF3 hallmark. Thus, the cause for this ChAT reduction is not due to distal detachment, on the contrary it might contribute to it.

On the other hand, we explore the existence of other metabolic ALS-related changes coexisting by 1 month of age. We observed the presence of mild oxidative stress and a marked early nuclear Tdp-43 accumulation in the MNs as concurrent early events to cholinergic reduction. Tdp-43 is involved in multiple steps of RNA metabolism, including transcription, splicing, or transport of several mRNAs. Interestingly, ChAT is one of the target genes of Tdp-43 (Buratti et al. 2010); thus, it might be directly involved in ChAT downregulation although extensive analyses should be performed to unravel this possibility. It is also interesting to highlight that Tdp-43 has normally observed mislocalized and aggregated in the cytoplasm in ALS samples from patients and in samples from late symptomatic SOD1^G93A^ mouse model, by 4 months of age (Cleveland and Rothstein 2001). We observed the starting delocalization to the cytoplasm by 3 months of age. Thus, Tdp-43 cellular localization changes might occur in parallel to dynamic metabolic changes that sequenced from early presymptomatic to late symptomatic stages. Therefore, detailed longitudinal studies should be considered to give further clues onto the etiopathogenesis of the diseases and to look for early biomarkers. In this regard, the concurrent mild oxidative stress early observed might be determinant to cause different molecular picture to that promoted by chronic or extensive oxidative stress which is presented later on. From our observations, we consider that the consequences of mild oxidative stress on Tdp-43 expression profile deserve further exploration considering its important impact on RNA metabolism of MNs and particularly to ChAT expression.

The early ChAT content reduction seems to have relevant consequences as we observed synaptic stripping-related events with loss of cholinergic innervations affecting the local circuitry at the spinal cord. Interestingly, we detected that ChAT content seems to accumulate abnormally in the perikaryon of MNs but diminished in processes and terminals from 2 months of age in the SOD1^G93A^ mice. These terminals were both afferent cholinergic boutons apposed to MNs and efferences from MNs to Renshaw cells. These observations are consistent with recent results reporting that ChAT can be sequestered in the soma because misfolded SOD1, present in the SOD1^G93A^ mice, impede particularly its axonal transport (Tateno et al. 2009). Therefore, first due to a downregulation and later on due to transport problems, ChAT is persistently reduced at the terminals and consequently lead to synaptic dysfunction that finally may end up with the cholinergic synaptic reduction. These events would affect the functionality of the local spinal cord circuitry. A reduced cholinergic input onto Renshaw interneurons may lead to less inhibition of synergic MNs to counteract the powerful effect of excitatory volleys brought about by glutamatergic stimulation. In consequence, MNs rest more vulnerable to excitotoxicity as it is characteristic in ALS (Pieri et al. 2003; Kuo et al. 2004). Moreover, dysfunction of Renshaw cells may precede the loss of glycinergic synapses onto MNs that was described for this mouse model to occur at symptomatic stage (Chang and Martin 2009). In agreement, alteration of this local circuitry has been also observed in ALS patients that present a decrease in recurrent inhibition (Raynor and Shefner 1994).

Our observations support the hypothesis that stripping of synaptic, in particular cholinergic, contacts with MNs might be one of the earliest events in ALS (Murray et al. 2010). Microglia has been reported to initiate stripping events (Blinzinger and Kreutzberg 1968), and MHC-I molecules are important for the balanced excitatory/inhibitory input synaptic withdrawal that normally occurs during development of the nervous system and in the synaptic removal process in axotomized MNs (Schutz 2005; Thams et al. 2008). We observed early activated microglia surrounding MNs concurrent with a loss of MHC-I expression within MNs. As the regenerative capacity is hampered in animals lacking MHC class I signaling (Thams et al. 2008), its early downregulation in the SOD1^G93A^ transgenic mice may influence both the unbalanced synaptic stripping and the difficulty in successful regeneration described in the model.

The diminution in cholinergic presynaptic boutons was also extended to changes in the postsynaptic sites, revealed by loss of Sig1-R immunoreactivity, an ion-buffering chaperone present in the subsurface cisternae of MNs underneath presynaptic C-boutons. Undoubtedly, this may influence ion channel conductivity and calcium buffering leading to changes in the pattern of MN firing and to altered motor behavior as that observed in the Sig1-R knockout mice (Mavlyutov et al. 2010). Reinforcing the potential etiopathogenic interest of the Sig1-R alteration is the recent discovery of Sig1-R mutations linked to some cases of MN disease (Luty et al. 2010).

Finally, how can an early and general reduction of cholinergic activity contribute to the different vulnerability observed between fast (more vulnerable) and slow MN types in ALS? One of the possibilities is that requirement of ACh production is markedly different between slow and fast MNs because they diverge in the total number of action potentials fired per day (Hennig and Lomo 1985) and in the amount of ACh released per action potential (quantal content). Thus, reduction of the presynaptic ChAT content may lead to decreased evoked ACh release, which would affect particularly the fast MNs. In contrast to the evoked potential, the rate of spontaneous ACh release is similar between fast and slow MNs (Reid et al. 1999). However, fast MNs are more dependent on endplate ACh receptor activation that acts as a retrograde signaling system for regulating their electrical properties, maintaining connectivity, and promoting regeneration (Reid et al. 1999). In this regard, a decrease in spontaneous ACh release would consistently weaken the strength of selective neuromuscular junction and hinder regeneration as observed in ALS (Murray et al. 2010).

## Conclusion

In conclusion, cholinergic dysfunction in the local circuitry of the spinal cord may be one of the earliest events in ALS pathogenesis. Thus, special interest in electrophysiological studies to perform repetitive nerve stimulation or analysis of recurrent inhibition to ascertain early ALS diagnosis in patients should be taken into consideration. Besides, the results presented herein suggest that ChAT production and function may be potential targets for therapy in ALS.
